# Vegetative versus Minimally Conscious States: A Study Using TMS-EEG, Sensory and Event-Related Potentials

**DOI:** 10.1371/journal.pone.0057069

**Published:** 2013-02-27

**Authors:** Aldo Ragazzoni, Cornelia Pirulli, Domenica Veniero, Matteo Feurra, Massimo Cincotta, Fabio Giovannelli, Roberta Chiaramonti, Mario Lino, Simone Rossi, Carlo Miniussi

**Affiliations:** 1 Neurology Unit, Azienda Sanitaria di Firenze, San Giovanni di Dio Hospital, Florence, Italy; 2 Cognitive Neuroscience Section, IRCCS Centro San Giovanni di Dio Fatebenefratelli, Brescia, Italy; 3 Neurology and Clinical Neurophysiology Section, Department of Neurological and Neurosensorial Sciences, Azienda Ospedaliera-Universitaria, Siena, Italy; 4 Rehabilitation Centre Villa alle Terme, Florence, Italy; 5 Department of Clinical and Experimental Sciences, National Institute of Neuroscience, University of Brescia, Brescia, Italy; Weill Cornell Medical College, United States of America

## Abstract

Differential diagnoses between vegetative and minimally conscious states (VS and MCS, respectively) are frequently incorrect. Hence, further research is necessary to improve the diagnostic accuracy at the bedside. The main neuropathological feature of VS is the diffuse damage of cortical and subcortical connections. Starting with this premise, we used electroencephalography (EEG) recordings to evaluate the cortical reactivity and effective connectivity during transcranial magnetic stimulation (TMS) in chronic VS or MCS patients. Moreover, the TMS-EEG data were compared with the results from standard somatosensory-evoked potentials (SEPs) and event-related potentials (ERPs). Thirteen patients with chronic consciousness disorders were examined at their bedsides. A group of healthy volunteers served as the control group. The amplitudes (reactivity) and scalp distributions (connectivity) of the cortical potentials evoked by TMS (TEPs) of the primary motor cortex were measured. Short-latency median nerve SEPs and auditory ERPs were also recorded. Reproducible TEPs were present in all control subjects in both the ipsilateral and the contralateral hemispheres relative to the site of the TMS. The amplitudes of the ipsilateral and contralateral TEPs were reduced in four of the five MCS patients, and the TEPs were bilaterally absent in one MCS patient. Among the VS patients, five did not manifest ipsilateral or contralateral TEPs, and three of the patients exhibited only ipsilateral TEPs with reduced amplitudes. The SEPs were altered in five VS and two MCS patients but did not correlate with the clinical diagnosis. The ERPs were impaired in all patients and did not correlate with the clinical diagnosis. These TEP results suggest that cortical reactivity and connectivity are severely impaired in all VS patients, whereas in most MCS patients, the TEPs are preserved but with abnormal features. Therefore, TEPs may add valuable information to the current clinical and neurophysiological assessment of chronic consciousness disorders.

## Introduction

Since the first description of the vegetative state (VS) [1], both the medical community and laypeople have considered the diagnosis of a VS important because of the implications in the end-of-life decision-making processes [2]. The distinctive feature of a VS is the dissociation between two basic elements of consciousness; *wakefulness* is intermittently maintained despite the lack of any behavioural signs of *awareness*.

In 2002, the term minimally conscious state (MCS) was introduced to describe patients who can be distinguished from VS patients by the presence of inconsistent cognitive behaviour that is either reproducible or sustained long enough to be differentiated from reflexive behaviour [3]. Despite these definitions, diagnosing a VS and distinguishing it from a MCS is a challenging task that is primarily based on the clinical history and the behavioural assessment of the patient. However, objective neurological evaluation is complicated by a series of confounding factors (e.g., motor impairment, fluctuating arousal, and sensory deficits) that make the judgment of awareness difficult and subjective, according to the clinician’s experience. Thus, erroneous diagnoses of VSs are common; the current rate of misdiagnosis is estimated to be greater than 40%, even in qualified centres [4]. Clinical misdiagnoses mainly involve classifying MCS as VS patients, which has consequences for the clinical management, rehabilitation strategies, prognosis, and caregiver expectations. These difficulties have encouraged clinical researchers to develop diagnostic techniques to complement the behavioural evaluations.

Neuroimaging methods (e.g., positron emission tomography – PET, and functional magnetic resonance imaging – fMRI) and electrophysiological techniques (e.g., electroencephalography –EEG, magnetoencephalography – MEG, and event-related potentials – ERPs) have revealed neocortical activation in several VS cases [5]–[14], suggesting the possibility of a partial preservation of cognitive processing, such as traces of speech comprehension and preserved motor imagery of complex tasks. Moreover, recent advances in structural MRI techniques, such as diffusion tensor imaging (DTI), which can determine the integrity of white matter tracts in vivo, have revealed the widespread damage of brain fibre tracts in VS patients [15]. Interestingly, most of the significant differences between VS and MCS patients tend to occur in the subcortical white matter and the thalamus [16]–[18]. These in vivo observations suggest a relevant disruption of both short- and long-range cortical connections, consistent with the neuropathological findings [19], [20], thus indicating that the VS (and, to a lesser extent, the MCS) is specifically associated with massive disruption of the thalamo-cortical and cortico-cortical connections. Therefore, the VS can be conceptualised as a global “cortical disconnection syndrome” [21]. Despite the unquestionable value of MEG, PET, fMRI, and DTI studies, these procedures cannot be performed at the bedside and are not universally available as a complement to the standard clinical evaluations.

Given these limitations, it has been advocated that alternative approaches should be considered to study brain functioning and network connectivity. In recent years, thanks to the implementation of new paradigms and new techniques for co-registering brain activity during brain stimulation (i.e., EEG with transcranial magnetic stimulation – TMS), it has become possible to study connectivity within brain networks [22]–[27].

This multimodal imaging approach has several advantages [22]–[28]. First, it allows the assessment of the local impact of TMS on neural processing through objective measurements of cortical reactivity, i.e., over the directly targeted area. Each TMS pulse excites cortical neurons directly as well as trans-synaptically [29] just below the stimulating coil, inducing a TMS-locked response that can be recorded by EEG, which is termed the TMS-evoked potential (TEP). This type of approach can be used to study the reactivity of a target area using the amplitude of the TEPs to test the overall state of the stimulated cortex. TEPs therefore reflect the direct activation of cortical neurons at the site of stimulation (i.e., cortical reactivity). TEPs are therefore considered quantifiable markers of the state of the brain that are directly generated and recorded from the cortex [30]. Second, TEP provides an assessment of the remote effects of TMS on neural processing in distal brain regions. Crucially, the local activation caused by the magnetic pulse (i.e., TEPs) spreads to connected areas trans-synaptically over the ensuing tens of milliseconds and can be traced by simultaneous EEG recording, which therefore reflects the rapid causal interactions among multiple groups of neurons (effective connectivity [31]) and not simply the temporal or coherence correlations. Cortical excitability and effective connectivity depend on the physiological state of the neurons in the stimulated cortex (state-dependency) and therefore vary as a function of the neuronal state. For example, it has been show that these measures are deeply modulated during the wakefulness/sleep cycle and during anaesthesia [32], [33]. TEPs are reproducible over time [34] and represent a non-invasive approach that has provided a new means of gaining valuable information about cortical reactivity and connectivity. Most importantly, the advantages of this method are that the efferent pathways and primary areas can be bypassed to deliver stimulation directly to the area of interest and that the procedure can be performed at the bedside without patient cooperation. Therefore, TEP appears to be an excellent tool for exploring cortical reactivity and tracking the connectivity of both the intrahemispheric and interhemispheric cortical networks in patients with disorders of consciousness, as recently demonstrated [35].

However, a direct comparison of TEP results with those obtained by more traditional bedside neurophysiological examinations, namely short-latency somatosensory evoked potentials (SEPs), ERPs, and spontaneous EEG is still lacking. SEPs, ERPs, and EEG have been extensively applied to study disorders of consciousness. Short-latency SEPs are recognised as a highly reliable test to identify patients with poor outcome (death or VS) in the acute phase following severe brain damage (for a review, see [36]). For example, the bilateral absence of median-nerve cortical SEPs is associated with no recovery in almost 100% of patients. ERPs (mainly the mismatch negativity and the P300 component) have been used extensively to detect the electrophysiological correlates of cognitive functions, potentially reflecting some level of awareness in VS and MCS patients [6], [10], [11], [13], [37]–[39]. The presence of ERPs has suggested some level of residual cognitive processing in a minority of VS and MCS patients, and ERPs are now recognised as valuable research techniques for diagnostic and prognostic purposes [40]. Measurements at the group level of the power spectra of EEG recordings under resting-state conditions have shown that the more slow the wave activity (delta band) observed, the lower the awareness level of the patient [6], [8], [41]. Resting-state EEG power spectra performed in the first month following severe brain injury can, to a degree, predict the chances of survival or death six months later in VS and MCS patients [42].

The aim of the current study was primarily to verify whether TEPs could differentiate VS patients from MCS patients. Our working hypothesis was that changes in the amplitudes and/or the scalp distribution of TEPs (i.e., smaller and more local responses), which reflect abnormalities in cortical reactivity and connectivity, might be more prevalent in VS patients than in MCS patients. A further aim was to compare the TEP results with those provided by traditional neurophysiological recordings (i.e., SEPs and ERPs) from the same patients.

## Materials and Methods

### Patients and Controls

This study included thirteen patients (4 females and 9 males between the ages of 25 and 89 years, with a mean age of 59 years) with chronic disorders of consciousness who had been classified according to internationally established criteria as being in a VS [43] or in an MCS [3]. Two additional patients were excluded due to excessive muscle artefacts during the EEG recording.

The aetiologies of the VS or the MCS were anoxia (n = 5), traumatic brain injury (n = 5), haemorrhage (n = 2), and hypoglycaemia-ischaemia (n = 1). Table 1 summarises the major demographic, clinical and, radiological information about all of the patients. The diagnoses were performed by multiple qualified examiners in a rehabilitation centre following a prolonged period of careful daily observation. In addition to the clinical evaluation, the JFK Coma Recovery Scale-Revised (CRS-R) was used for the behavioural assessment of the patients [44] and was also performed on the same day as the TMS-EEG recording (see Table 2 for the subscale scores for each patient). All MCS patients (except MCS3) showed only non-reflex movements, such as localising noxious stimuli or visually pursuing a moving or salient stimulus, and were qualified as MCS minus (MCS-), which indicates minimal levels of behavioural interactions [45]. None of the patients had a history of neurological disease prior to their coma. Patients did not receive sedating drugs within the 24 h preceding the recordings, but three (2 VS, 1 MCS) were taking antiepileptic medications that could not be withdrawn. The time that had elapsed between the injury and the neurophysiological recordings ranged from 7 to 65 months (mean 31.8 months). The proportions of anoxic and traumatic injuries were similar in the two groups (see Table 1).

**Table 1:** Summary of demographic, clinical and neuroimaging data.

Patient	Age/Sex	Aetiology	Months post-injury	CRS-R	CT/MRI findings
VS1*	25/m	TBI	14	4	Left subdural haematoma, bilateral hydrocephalus
**VS2**	65/m	Hypoglycaemia/ischaemia	07	6	Diffuse bilateral leuco-encephalopathy
VS3*	29/m	Anoxia	33	5	Diffuse atrophy
**VS4**	70/m	TBI	47	6	Left temporal lesion, diffuse atrophy
**VS5**	89/f	Haemorrhage	56	7	Left parietal haemorrhage, right periventricular white matter lesions
**VS6**	71/m	Anoxia	41	4	Diffuse atrophy, aneurysm of left middle cerebral artery
**VS7**	67/f	TBI	12	4	Left temporal haematoma, bilateral hydrocephalus
**VS8**	74/m	Anoxia	08	6	Diffuse atrophy
**MCS1**	41/m	Anoxia	65	9	Diffuse atrophy, pontine ischaemic lesion
**MCS2**	62/m	TBI	08	9	Left temporal haematoma
MCS3*	54/f	Haemorrhage	59	12	Right frontal haemorrhage
**MCS4**	63/m	TBI	56	10	Bilateral frontal contusions
**MCS5**	58/f	Anoxia	08	9	Periventricular white matter lesions

Abbreviations: MCS = minimally conscious state; VS = vegetative state; Age in years; Sex m = male; f = female; TBI = traumatic brain injury; CRS-R = Coma Recovery Scale-Revised; * = daily treatment with antiepileptic drugs: VS1, phenobarbital (50 mg); VS3, oxcarbazepine (900 mg); MCS3, phenytoin (300 mg).

**Table 2:** Coma Recovery Scale-R, subscales and total scores for all patients.

Patient	Auditory function	Visual function	Motor function	Oromotor/Verbal function	Communication	Arousal	Total score
**VS1**	Startle	Startle	Abnormal posturing	None	None	With stimulation	4
**VS2**	Startle	Startle	Flexion withdrawal	None	None	Without stimulation	6
**VS3**	Startle	Startle	Flexion withdrawal	None	None	With stimulation	5
**VS4**	Startle	Startle	Flexion withdrawal	None	None	Without stimulation	6
**VS5**	Startle	Startle	Flexion withdrawal	Oral reflexive movement	None	Without stimulation	7
**VS6**	Startle	None	Abnormal posturing	Oral reflexive movement	None	With stimulation	4
**VS7**	None	None	Flexion withdrawal	None	None	Without stimulation	4
**VS8**	None	Startle	Flexion withdrawal	Oral reflexive movement	None	Without stimulation	6
**MCS1**	Startle	Fixation	Localization to noxious stimulation	Oral reflexive movement	None	Without stimulation	9
**MCS2**	Localization	Visual pursuit	Flexion withdrawal	None	None	Without stimulation	9
**MCS3**	Localization	Visual pursuit	Localization to noxious stimulation	Vocalization/oral movement	None	Without stimulation	12
**MCS4**	Startle	Visual pursuit	Localization to noxious stimulation	Oral reflexive movement	None	Without stimulation	10
**MCS5**	Startle	Fixation	Localization to noxious stimulation	Oral reflexive movement	None	Without stimulation	9

A group of five healthy volunteers (1 female and 4 males between the ages of 24 and 43 years) served as the controls.

Written informed consent was obtained from the healthy volunteers and from each patient’s legal surrogate, according to the Code of Ethics of the World Medical Association (Declaration of Helsinki). The experimental protocol was performed in accordance with the ethical standards and was approved by the Ethical Committee of the IRCCS Centro San Giovanni di Dio Brescia.

### Neurophysiological Procedures

All patients and control subjects underwent the same neurophysiological protocol, which consisted of two TEP sessions; the first session involved the TMS, and the second session involved sham stimulation. In the sham condition, a 30-mm-thick plywood shield, built to appear as an integral part of the apparatus, was interposed between the coil itself and the scalp, separating the two [46], [47] (see [48] for sham stimulation details ). In one additional session, the SEP and ERP recordings were performed.

### Transcranial Magnetic Stimulation

TMS was applied to the scalp overlaying the primary motor cortex (M1) of the less-affected hemisphere of the patients and to the dominant hemisphere of the controls. The less-affected hemisphere was chosen based on clinical and neuroradiological examinations. TMS was never applied to overt cortical lesions (according to CT/MRI findings).

The entire experimental session lasted approximately 60 min and was conducted at the bedside for all 13 patients. The experimenters paid special attention to having the patients awake, with their eyes open, throughout the recording sessions. The recordings from the control subjects were taken while each subject was lying in a reclining armchair.

TMS was delivered using a Magstim Super Rapid stimulator and a double 50-mm figure-eight coil (Magstim Company, Whitland, UK). The coil was placed tangentially to the scalp with the longer axes perpendicular to the central sulcus over M1. To establish the optimal position (hot-spot) for eliciting motor-evoked potentials (MEPs) in the contralateral first dorsal interosseous (FDI) muscle, the coil was moved forward in approximately 0.5 cm steps along the fronto-central region of the scalp. After the target area was identified, the coil was stabilised with a mechanical support, which consisted of a holding arm (Magic Arm, Manfrotto, Cassola, Italy) and two large clamps fixed to the head of the bed. Once the coil was immobilised, we determined the actual resting motor threshold (RMT), which was defined as the lowest stimulus intensity that produced at least five surface-recorded MEPs of 50 µV with a 50% probability in the FDI muscle [49]. If no MEP could be elicited (two VS and one MCS), we used the 10–20 International System to locate M1, and the stimulated site corresponded to the C3/C4 location. To achieve comparable stimulation, all patients and control subjects were stimulated using 75% of the maximum stimulator output [26]. After positioning the coil over the hot-spot, a total of 400 single TMS pulses were applied (200 for real stimulation and 200 for sham stimulation). The TMS pulses were applied at random interstimulus intervals ranging from 0.25 to 0.5 Hz. The parameters that were used in this study were in accordance with international safety recommendations [50].

### EEG Recordings and Analysis

TMS-compatible EEG equipment (BrainAmp 32 MRplus, BrainProducts GmbH, Munich, Germany) was used to record the TEPs from the scalp. The EEG signal was continuously acquired from 19 scalp electrodes (Fp1, Fp2, F7, F3, Fz, F4, F8, T3, C3, Cz, C4, T4, T5, P3, Pz, P4, T6, O1, and O2) that were mounted on an elastic cap and placed according to the 10–20 International System. The ground electrode was positioned on Fpz, and the linked mastoids served as the reference. The signal was amplified, bandpass filtered at 0.1–1,000 Hz, digitised at a sampling rate of 5,000 Hz and stored for offline analysis. We used TMS-compatible Ag/AgCl-sintered ring electrodes. The horizontal and vertical eye movements were detected by recording an electrooculogram from two pairs of electrodes located to the left and right of the external canthi and on the supraorbital and infraorbital ridges of the right eye. The skin/electrode impedance was maintained at below 5 kΩ.

The resting EEG recorded in each patient at the beginning of the sham session was analysed by Fast Fourier transform to calculate the power of the frequency spectra (for details of the EEG spectral analysis, see the Material S1).

### TEP Processing and Analysis

EEG recordings were processed using the Brain Vision Analyser (BrainProducts GmbH, Munich, Germany). After the data collection, the continuous EEG signals were divided into 500 ms epochs spanning from 100 ms before to 400 ms after the TMS delivery. Each epoch was baseline-corrected using the pre-TMS pulse interval, and all of the epochs were visually inspected to exclude those contaminated by excessive background noise and eye movements (blinks or saccades exceeding ±75 µV). To maintain an acceptable signal-to-noise ratio, we set a lower limit of 70 artefact-free trials per subject per condition. In some patients (VS6, VS8, MCS5), independent component analysis [51] was applied to remove 50 Hz interference and/or muscle activity artefacts from the signal before averaging the remaining trials. The 20 ms interval immediately following the TMS pulse was excluded from further analyses to avoid an initial artefact caused by the currents induced by the magnetic field and the eventual TMS-evoked muscular scalp responses [52]. Furthermore, to exclude the possibility that the TEPs were due to auditory potentials evoked by the click associated with the TMS discharge, the EEG epochs obtained during the sham condition were subtracted point-by-point from those obtained during the real TMS. In this regard, it has been previously demonstrated that the auditory potentials evoked by the clicking associated with the TMS do not significantly alter the TEP recording [53]. Moreover, it should also be considered that this condition was equally present in all subjects.

Due to the originality of this TMS-EEG approach, we had no *a priori* hypothesis regarding the TEP components that were likely to be absent or modified. Therefore, the data analysis was performed using the measurements of the voltage value over successive time bins from the closest electrode to the hot-spot (ipsilateral) and from the contralateral electrode to the hot-spot (C3 or C4). To detect the significant TEPs in the averaged signal, the peaks were visually identified and validated through a statistical analysis as local maxima or minima; i.e., peaks were identified when the positive or negative responses measured with respect to the 100 ms preceding the TMS pulse for a minimum of 100 consecutive sampling points (equivalent to 20 ms) exceeded three times the standard deviation of the baseline, which is in line with previous studies (for similar data analysis, see [54]). We tested for a normal distribution of the baseline data for each patient by performing a Kolmogorov–Smirnov test (for all P>0.2) considering each data point included in the baseline period.

Furthermore to test for significant difference between patients and healthy controls, EEG signal recorded from nine electrodes (F3, Fz, F4, C3, Cz, C4, P3, Pz, P4) was rectified and mean area values were calculated for each patient and each control for significant time windows, as revealed by the previous analyses on voltage value over successive time bins. The area values of each interval was submitted to a separated ANOVA with group as between factor and region (ipsilateral to TMS, contralateral to TMS, midline) and electrode as within factor. Post-hoc tests were performed by means of Tukey’s HSD.

### Source Localisation Analysis

To individualise the way in which the TMS-induced responses propagated over the scalp, we used standardised low-resolution electromagnetic tomography (sLORETA) [55], which provides estimates of the cortical sources of the evoked potentials. LORETA was used to compute 3D linear solutions (LORETA solutions) for the EEG inverse problem within a three-shell spherical head model, including the scalp, skull and brain compartments. The cortical source was only estimated for the significant TEPs of each patient.

### SEP Recording and Analysis

The short-latency (100 ms post-stimulus analysis interval) SEPs from right and left median nerve stimulations of the wrist (electrical stimuli, duration 0.3 ms, frequency 3 Hz, with intensity adjusted to produce a visible thumb twitch) were recorded with a four-channel montage (Erb’s point ipsilateral-Erb’s point contralateral to the stimulation site, Cv6-AC, CP3or CP4-Ear ipsi, CP3 or CP4-Fpz) according to the recommendations of the International Federation of Clinical Neurophysiology [56]. The baseline-to-peak amplitude of the N20 deflection recorded over CP3 or CP4 was classified as normal, abnormal (<0.7 µV) or absent. When the SEPs were asymmetric over the two hemispheres, the better response was selected for analysis [10]. Eighteen normal subjects served as controls.

### ERP Recording and Analysis

ERPs were obtained by means of a simple “oddball” paradigm using auditory stimulation. Three successive series of two randomly intermixed tones (1000 Hz, overall probability 80%; 2000 Hz, overall probability 20%) were delivered binaurally through earphones at a rate of one tone (90 dB SPL, 50 ms plateau time, 2 ms rise and fall slope) every 1.1 s.

Each patient was asked to keep a mental count of the rare (target) tones while ignoring the frequent (non-target) tones. This was conducted independently of patients’ language comprehension abilities and active participation in performing the task [6], [11], [13], [37]. EEG activity was continuously recorded (bandpass filtered at 0.1–100 Hz; digitised at a sampling rate of 1024 Hz) from 19 scalp electrodes mounted on an elastic cap (10–20 International System) all referred to linked earlobes, and the ground electrode was positioned on Fpz. Skin/electrode impedance was maintained below 5 kΩ. Eye movements were detected by recording an electro-oculogram from a pairs of electrodes located on the nasion and the left zygomatic bone.

Trials containing artefacts exceeding ±100 µV were automatically rejected from the averages. Responses to frequent and rare tones were averaged separately over 1000 ms, including a 200 ms pre-stimulus interval. After averaging, a further low-pass filter at 20 Hz was applied. Amplitudes were measured relative to the pre stimulus baseline. Wave detection (N1, P3) was achieved by visual identification from a trained neurophysiologist, based on latency (N1∶80–150 ms; P3∶280–500 ms) and scalp topography (frontal-central for N1; central to parietal for P3). A waveform (N1, P3) was considered present when appropriate latency and topography were observed [57]. In addition, the averaged responses to frequent and rare tones were compared statistically by applying a t-test on a sample-by-sample basis using a software package for neurophysiological analysis (NPX Lab 2011, ERP module; www.brainterface.com). Differences between frequent and rare ERPs were considered significant when p<0.001. ERPs in patients were compared with those obtained in a group of 25 age-matched healthy controls.

### Comparison Analysis

With the aim of evaluating the congruency of the results between different neurophysiological measures in VS and MCS populations, the χ2 test with Yates correction for small samples was used. In this analysis, the direct proportions from the TEPs results were compared with those obtained by SEPs and ERPs.

## Results

### TMS-EEG

**Figure 1:**
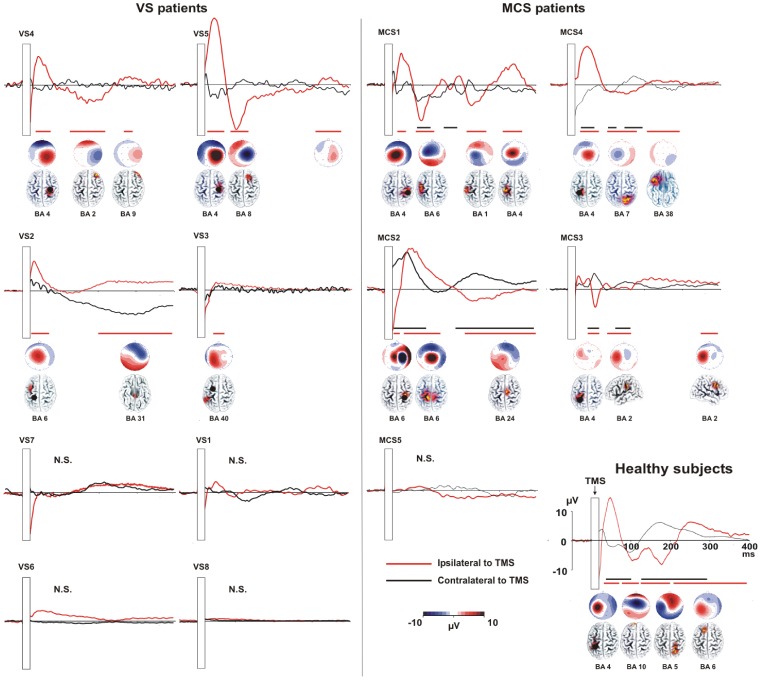
TEPs recorded in VS patients, MCS patients and healthy controls (grand average of five individuals). The figure presents the TEPs recorded from C3 and C4 following stimulation of the left or right M1 in VS (left insert) and MCS patients (right insert). The right lower part of the figure indicates the grand average TEPs obtained in five healthy controls stimulated above the left primary motor cortex (M1; C3). For all patients and the control group, the presented TEPs were recorded from the closest electrode to the hot-spot (ipsilateral to TMS – red line) and from the corresponding electrode on the contralateral hemisphere (black line). The hot-spot is indicated with a black dot. The responses obtained during the sham condition were point-by-point subtracted from those obtained during the real TMS. The significant time-windows (i.e., EEG signal exceeding three times the standard deviation of the pre-stimulus activity for at least 20 ms) are separately indicated for the ipsilateral electrode with a horizontal red line and for the electrodes contralateral to the TMS hot-spot with a black line.

TEPs were recorded in all of the patients and normal subjects. After artefact rejection, between 75 and 198 out of 200 epochs were used to determine the average TEP response. In the normal subjects, the TMS pulse evoked a sequence of statistically significant deflections with alternating positive and negative polarities (Figure 1). The TEPs persisted for the entire analysis epoch and spread from the stimulated site (local reactivity) to the connected areas in the same hemisphere, then to the contralateral hemisphere (long-range connectivity) (see Figure 1).

#### Temporal interval results

The temporal intervals during which the evoked responses reached statistical significance followed the TMS pulse by 28–80 ms, 90–200 ms and 220–400 ms (black and red lines in Figure 1).

In the MCS group (n = 5), four patients (MCS1, MCS2, MCS3 and MCS4) manifested significant activation in both the ipsilateral and contralateral hemispheres relative to the TMS site. The fifth MCS patient (MCS5) did not exhibit any significant cortical activation in either the ipsilateral or the contralateral hemisphere.

**Figure 2:**
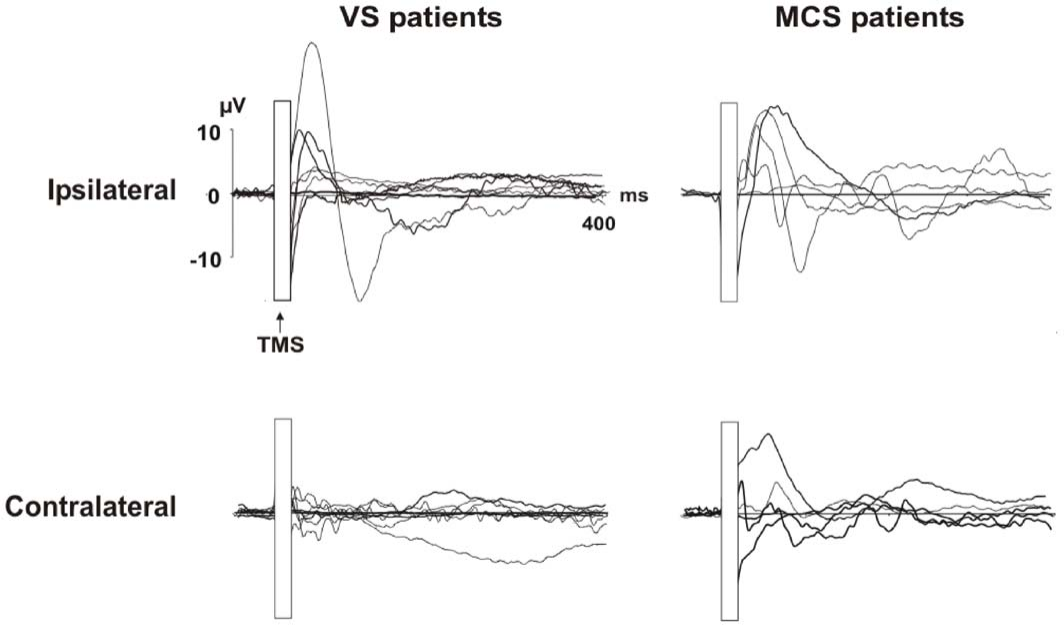
Individual TEPs recorded from both hemispheres for the two groups of patients: VS patients are shown in the left column, and MCS patients are shown in the right column. TEPs recorded over the stimulated hemisphere are displayed in the upper row, and TEPs recorded over the contralateral hemisphere are in the lower row.

In the VS group (n = 8), three patients (VS2, VS4 and VS5) exhibited responses to the TMS pulse in the stimulated hemisphere (Figures 1 and 2) but no significant activation in the contralateral hemisphere. In the remaining five VS patients, no significant activation was identified locally or distal to the stimulation site (VS1, VS3, VS6, VS7, and VS8) (Figures 1 and 2).

#### Mean area results

ANOVAs performed on TEP mean area in these significant intervals revealed that in all consciousness disorder patients, the TEPs were clearly different from those recorded in the healthy controls. As shown in Figures 1 and 2, the healthy controls manifested complex patterns of responses (i.e., TEPs) that were not present in the patients.

When considering the first interval (28–80 ms) analyses showed a significant group by region by electrode interaction (F8, 64 = 3.93; p<0.00). Post-hoc tests confirmed that the response in the stimulated area was significant greater for healthy controls than for MCS and VS patients (all comparisons p<0.01). In the healthy control group the electrode close to the stimulation site (C3) was characterized by a greater activation when compared to all other electrodes (all comparisons p<0.05). Moreover MCS patients showed also a larger activity over the stimulated area (C3 or C4) when compared to the activity recorded from all other electrodes (all p<0.05). No significant modulation was found within VS group (all p<0.05).

Analyses on the second interval (90–200 ms) showed significant group by region (F4,32 = 3.92; p<0.01) and group by electrode (F4,32 = 6.67; p<0.001) interactions. The group by region effect was explained by a larger activity of the central regions (Fz, Cz, Pz) in healthy control when compared to VS and MCS groups (all comparisons p<0.05). No other significant effects were found. Group by electrode significant interaction indicated that in healthy controls there was a stronger activation over frontal electrodes when comparing area values to other electrodes within and between groups (all comparisons p<0.05).

When considering the last interval (220–400 ms) analyses showed a significant group by region by electrode interaction (F8,64 = 13.7; p<0.00). Also in this case, C3 was characterized by a greater activation in healthy control when compared to all other electrodes within the group (all comparisons p<0.001) and to all the electrodes for MCS and VS groups (all comparisons p<0.05). Interestingly the same results were found for P3 (all comparisons p<0.01), thus indicating that in normal subjects there was a spreading of activation towards parietal cortex. No other effects were found within the patient groups.

#### Source localisation results (sLORETA)

The cortical source was only estimated for the significant TEPs of each patient. The sLORETA analysis confirmed what had previously been demonstrated by the descriptive analysis. The MCS patients (i.e., MCS1, MCS2, MCS3 and MCS4) were characterised by bilateral activation of the TMS-targeted area. Specifically, activation was observed in Brodmann areas 4, 6 and 7, which correspond to M1, the premotor cortex and the somatosensory association area, respectively. In addition, we observed activation in the homologous contralateral areas.

Three VS patients (i.e., VS2, VS4 and VS5) were characterised by activation close to the stimulated area (BA 4 and 6), but the activation did not propagate to the hemisphere contralateral to the TMS.

Overall, as revealed by statistical analyses on TEPs, by the topographical distribution and by sLORETA analysis; in the control subjects, the TMS elicited a spread of activation towards the near and distant cortical areas with respect to the stimulated area, which was not present in the VS or MCS patients. In the MCS patients, we observed some significant ipsilateral and contralateral activation, but this activation was significantly different from that detected in the controls (i.e., reduced TEP morphology characterised by smaller amplitude and smaller temporal windows of significance). Moreover, when a partial spread of activation was present, it was confined to the contralaterally stimulated homologous area (i.e., M1).

The patients were grouped into three patterns (Table 3) based on the presence/absence of TEPs in the hemispheres ipsilateral (local reactivity) and contralateral (long-range connectivity) to the site of TMS stimulation; the “Bilateral” pattern included patients with both ipsilateral and contralateral activation, the “Ipsilateral” pattern included those with activation of only the ipsilateral hemisphere, and the “None” pattern included patients exhibiting neither ipsilateral nor contralateral hemispheric activation.

**Table 3:** Summary of neurophysiological data.

Patient	TEPs	SEPs	ERPs N1	ERPs P3	EEG background activity
**VS1**	None	a/a	p	a	Bilateral theta
**VS2**	Ipsilateral	abn/abn	p	a	Low amplitude theta, fast rhythms
**VS3**	None	na/na	p	a	Bilateral delta-theta
**VS4**	Ipsilateral	abn/a	p	a	Bilateral theta
**VS5**	Ipsilateral	n/abn	p	a	Bilateral delta-theta, fast rhythms, dysrhythmia
**VS6**	None	abn/abn	a	a	Low amplitude, bilateral delta
**VS7**	None	n/abn	p	a	Bilateral delta, intermittent
**VS8**	None	abn/abn	p	a	Low amplitude theta
**MCS1**	Bilateral	abn/n	p	a	Low amplitude bilateral theta, intermittent alpha; fast rhythms
**MCS2**	Bilateral	n/a	p	a	Bilateral delta-theta
**MCS3**	Bilateral	a/n	p	a	Bilateral frontal theta, intermittent posterior alpha
**MCS4**	Bilateral	a/a	p	a	Bilateral delta-theta
**MCS5**	None	abn/abn	a	a	Bilateral theta-alpha, dysrhythmia

Bilateral = presence of ipsilateral and contralateral TEPs; Ipsilateral = presence of ipsilateral TEPs only; None = bilateral absence of TEPs. Abbreviations: a = absent; abn = abnormal; n = normal; p = present; na = not available.

The correlation between the clinical assessment (VS or MCS) and the TEP classification was significant. Namely, the absence of contralateral TEPs significantly discriminated between the VS and MCS groups (χ2 = 5.870, p<0.015). In contrast, no significant difference in the presence/absence of ipsilateral TEPs emerged between the two groups of patients (χ2 = 0.853, p<0.356). Thus, the analysis of the local reactivity and the intracortical connectivity, as reflected by the amplitudes and scalp distributions of the TEPs, discriminated between VS and MCS patients in 92% of the cases (12 of 13 patients).

### SEP Results

**Figure 3:**
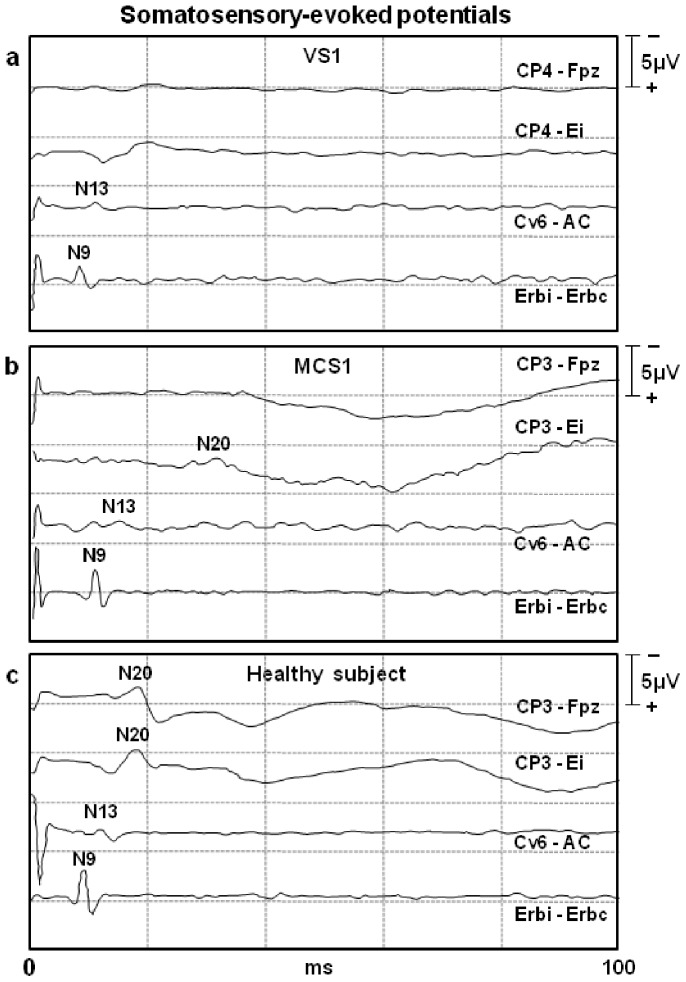
Representative short-latency median nerve somatosensory-evoked potential (SEPs) tracings in one VS1 (a) patient, one MCS1 (b) patient, and one normal (c) subject. In the VS patient, the electrical stimulation at the wrist elicited the activation of the brachial plexus at Erb’s point (wave N9) and of the cervical spine (wave N13) with normal amplitude/latency characteristics. Even the subcortical far-field P14 wave was recordable, while no cortical activation was detectable (i.e., absence of N20 wave) in the contralateral somatosensory cortex of the VS1 patient. In patient MCS1, the SEPs were abnormal, as the cortical component N20 was delayed and of a very small amplitude (<0.7 µV) and was only detected with the earlobe-reference montage. CP3/CP4: parietal scalp electrodes contralateral to stimulation; Fz: reference mid-frontal scalp electrode; Ei: reference electrode on the earlobe ipsilateral to stimulation; Cv6: posterior spinal cervical electrode over the 6th cervical spinous process; AC: anterior neck reference electrode; Erbi/Erbc: clavicle Erb’s point electrodes, ipsilateral or contralateral to stimulation.

The N20 was normal in five patients (2 VS, 3 MCS), abnormal in five (4 VS, 1 MCS) and absent in two (1 VS, 1 MCS) (Table 3, Figure 3). No significant difference in the N20 peaks between the two groups of patients was observed (χ2 = 0.245, p = 0.621).

### ERP Results

**Figure 4:**
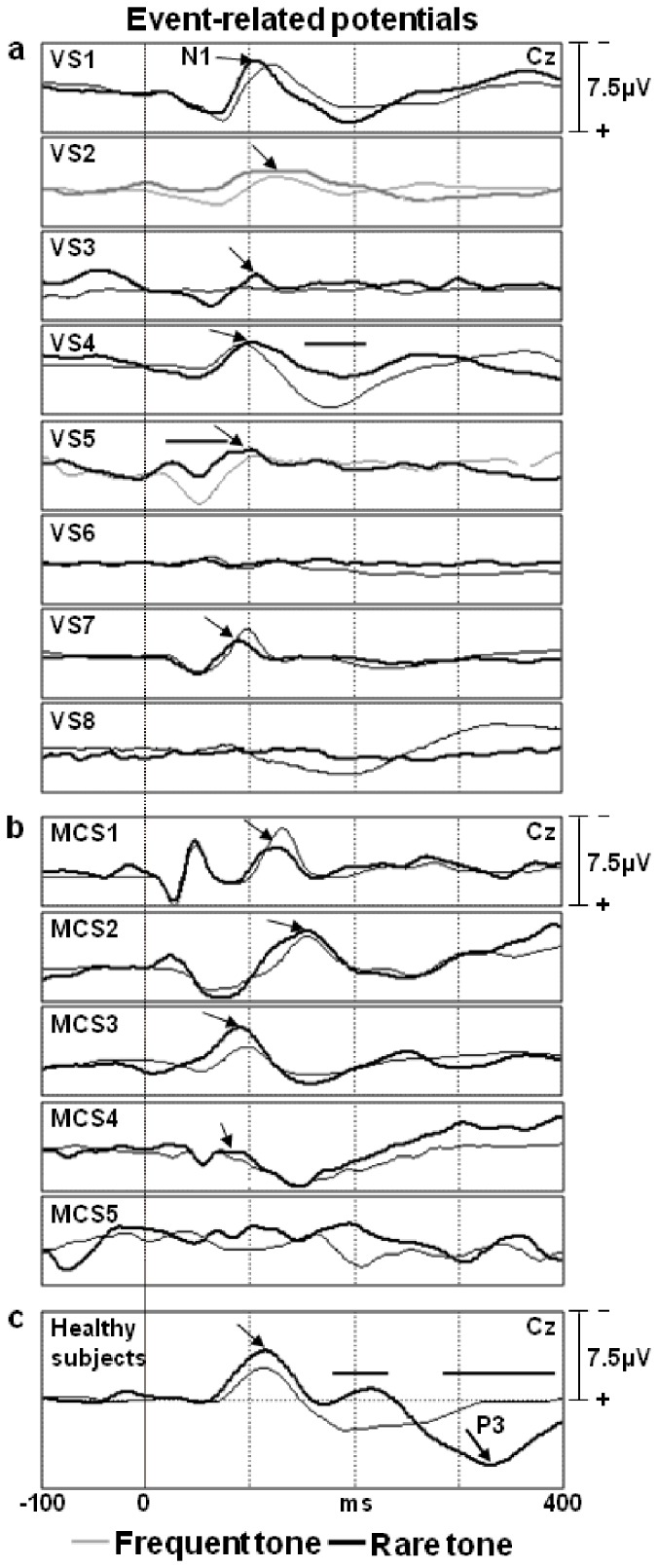
Individual event-related potentials (ERPs) recorded from the Cz electrode to frequent (thin line) and rare (thick line) tones. Panel **a**: vegetative patients (VS). Panel **b**: minimally conscious patients (MCS). Panel **c**: normal control subjects (grand average of 25 individuals). Horizontal segments refer to the time windows where the ERP responses to frequent and rare tones differed significantly (sample-by-sample t-test, p<0.01).

The N1 was present in eleven patients (7 VS, 4 MCS) and absent in two (1 VS, 1 MCS), but no significant correlation emerged between the presence/absence of the N1 and the clinical assessment of VS or MCS. (χ2<0.001, p = 1.000) (Table 3 and Figure 4). The mean latencies of the N1 at the Cz electrode were 106 ms (SD 15 ms) for VS patients and 116 ms (SD 21 ms) for MCS patients (for healthy controls, the mean was 107 ms, SD 11 ms). The mean amplitudes of the N1 at Cz were 4.4 µV (SD 2.4 µV) for VS patients and 6.0 µV (SD 3.8 µV) for MCS patients (for healthy controls, the mean was 8.5 µV, SD 3.4 µV).

A significant difference in the amplitudes between the ERP responses to frequent and rare tones emerged for only two patients (VS4, VS5) at rather early time intervals of their waveforms (below 200 ms) (t-test p<0.01) (Figure 4). The P3 was not observed in any patients.

### EEG Power Spectra Results

The EEG frequency power spectra did no significantly differ between the MCS and VS patients for any of the frequency bands considered in the analysis (all Fs smaller than 2.19, P>0.79). See Supplementary material Figure S1.

## Discussion

This TMS-EEG study represents an attempt to electrophysiologically interrogate, at the bedside, the cortical reactivity and connectivity in patients with chronic disorders of consciousness, classified as either in a permanent VS (according to the Multi-Society task Force on PVS [43]) or in a long-lasting MCS. Because there was no chance of neurological recovery in our VS patients, this study should not be regarded as having prognostic implications. Instead, it represents an investigation into the possibility of differentiating between VSs and MCSs using a new neurophysiological technique (i.e., the combination of TMS and EEG, which allows for the acquisition of TEPs) [24], [54]. This study also aimed to compare the diagnostic indications of TEPs with those of more traditional neurophysiological investigations, such as SEPs and ERPs.

As expected, the TEPs were abnormal in all of the VS and MCS patients compared with those of the healthy controls. However, the TEP patterns (Table 3) in the VS patients were significantly different from those observed in the MCS patients; in four of the five MCS patients, the TEP distribution over the scalp involved both hemispheres, although the responses were of clearly reduced amplitudes compared with those in the healthy subjects. In contrast, in the VS patients, the responses were confined to the stimulated hemisphere in three patients and were completely undetectable in five patients. TEPs have been hypothesised to originate from the direct stimulation of the cortex underlying the TMS coil (local reactivity) and from the ensuing activation of the ipsilateral and contralateral cortical areas via specific short- and long-range intrahemispheric and transcallosal connections [22], [23], [54]. Therefore, our results suggest that the cortical connectivity and local reactivity were severely impaired in the majority of the permanent VS patients, whereas they were much less impaired in most of the MCS cases. Notably, all of the VS patients lacked activation of the hemisphere contralateral to the focal TMS pulse, consistent with the hypothesis that consciousness primarily depends on the activation and rapid interaction of widely distributed cortical networks [32], [58]. Once the cortical connectivity between the networks is severely disrupted (as was the case in our permanent VS patients), consciousness fades.

One could argue that TMS might have failed to activate cortical neurons due to the presence of discrete lesions or cortical atrophy in the stimulated area. However, this possibility is unlikely because CT/MRI findings excluded the presence of relevant morphological alterations under the TMS coil site. In addition, the local (under the coil) suppression (amplitude below 10 µV) of background EEG was never detected in our patients, even in those not exhibiting TEPs. This reduces the likelihood of non-viable cortex (marked atrophy) existing under the stimulating coil. Finally, the TMS over M1 elicited a motor evoked potential (MEP) in the contralateral FDI muscle of most patients, thus providing evidence that the corticospinal neurons were actually responsive to TMS. This occurred even in three out of the five VS patients in which the TEPs failed to reveal local cortical reactivity (VS6, VS7, and VS8). In contrast to the other VS patients, the two VS patients in whom TMS did not elicit MEPs (VS1 and VS3) were treated with antiepileptic drugs (AEDs). Theoretically, AEDs may also increase the threshold of cortical reactivity and prevent the cortical spread of TEPs [59]. However, MEPs were also absent in the single AED-treated MCS patient (MCS3) despite the preservation of ipsilateral and contralateral TEPs (Table 3). This strongly supports the view that the absence of TEPs is not strictly related to the absence of MEPs and that AED treatment alone cannot explain such markedly different TEP patterns between the VS and MCS groups. Among the five MCS patients, only one (MCS5) manifested a bilateral absence of TEPs. In this patient, no ERP components (N1, P3) could be elicited. Interestingly, the same neurophysiological pattern (no TEPs, no ERPs) was observed in the vegetative patient VS6, and both patients had an anoxic aetiology. Therefore, the disruption of the cortico-cortical connectivity subserving the TEPs and ERPs appears to be more severe in anoxic patients, irrespective of the clinical diagnosis. This finding also suggests that more deteriorated (None or ipsilateral patterns only) TEP profiles are highly suggestive of a VS condition, but such an association cannot be considered absolute. Following this reasoning, the cortical connectivity tracked by TEPs might not specifically reflect the neural circuitry (whatever it is) underpinning consciousness; rather, this connectivity would relate to complex networks functionally connecting different cortical areas and likely subserving a variety of cortical activities.

Thus far, only one other study [35] has examined disorders of consciousness with TMS-EEG. This study was performed on a group of 17 patients (10 VS, 5 MCS, 2 locked-in patients). In the VS group, TMS triggered only ipsilateral responses or no responses at all, while MCS patients exhibited bilateral responses that spread over both hemispheres. In addition, the recovery from VS into MCS was associated with the bilateral recovery of TMS-EEG responses. Despite the fact that most of the patients reported by Rosanova and colleagues [35] were in the early phase of their condition (12 to 172 days after the insult), the findings in our patients with long-term disorders of consciousness are quite concordant with those obtained in that study and therefore add to the generalisability of the method. Thus, the TMS-EEG technique can be useful in differentiating VS from MCS and could complement the behavioural assessment during the diagnostic workup, thereby reducing the high probability (almost 40% in qualified centres) of clinical misclassification [4].

In a novel approach, we compared the TMS-EEG results with those obtained by other neurophysiological methods (i.e., short-latency SEPs, ERPs) in the same patients. In this sample of patients, TMS-EEG proved superior to these more traditional techniques in differentiating VS from MCS. This result should not be regarded as completely unexpected. Indeed, short-latency SEPs, which explore the oligosynaptic dorsal column-lemniscal system, successfully predict a negative outcome in comatose states [36] but exhibit insufficient accuracy in classifying patients with chronic disorders of consciousness [10], [38]. The ERP components N1 and P3, elicited in an oddball paradigm, reflect the sequential activation of multiple cortical and subcortical generators [60] and are associated with cognitive processes requiring selective attention and the updating of working memory. However, the many ERP studies conducted to differentiate between VS and MCS patients have not provided unequivocal results. Some studies have reported evidence of different ERP patterns between the two groups of patients [37]–[39], whereas others have demonstrated that the ERPs did not significantly differ between the VS and MCS patients [6], [10]. The latter studies examined patients with long-term conditions (in contrast to the former studies), which could possibly explain their negative results. Our VS and MCS patients were also in chronic conditions; the P3 was not observed in any patients, and the ERPs did not differentiate between individuals belonging to the two diagnostic groups. A very prolonged state of impaired consciousness, either VS or MCS, is the consequence of a more severe brain injury, and these conditions may be represented by more deteriorated ERP profiles.

It should be stressed that TEPs and ERPs explore different aspects of brain function. On the one hand, TEPs directly assesses the basic properties of complex intra- and inter-hemispheric cortical circuitries, such as the reactivity/excitability, effective connectivity and the balance between inhibition and facilitation [61]. On the other hand, ERPs are subtended by widespread brain systems that involve the activation of associative cortices and are specifically linked to cognitive processes [57]. Therefore, the two techniques explore different neural circuitries to provide complementary information, and whenever possible, these methods should be applied together in the study of chronic disorders of consciousness. Finally, the background EEG spectral analysis also did not significantly differ between the MCS and VS patients, consistent with a previous study [62].

The current TEP results and those of Rosanova and collaborators [35] concur to indicate that simultaneous TMS-EEG recording is a powerful procedure for identifying individuals with preserved awareness among those patients with chronic disorders of consciousness. In our study, this technology very efficiently distinguished patients with VS from patients with MCS who exhibited only minimal levels of behavioural interactions (categorised as MCS minus). Unlike Rosanova et al. [35], our EEG recordings were conducted in a rehabilitation center where patients were staying over a prolonged period of time. These findings, on long-term patients, show that absent or localized TMS-EEG activation is associated with worse outcomes as compared to bilateral activation, and this could have strong implications for the choice of rehabilitation strategy. In addition, a major advantage of TMS-EEG is that it measures the cortical responses that can reflect consciousness independently of muscle function. This feature is of paramount importance in the evaluation of brain-injured patients with severe motor impairment. However, it needs to be mentioned that TMS-EEG requires special amplifiers to overcome the large artefacts induced in standard EEG because of the strong magnetic field created by TMS, which might represent a technical limitation [63]. Also, the possibility of false negatives (such as MCS5 in our case) cannot be underestimated, and this potential warrants further studies involving larger numbers of patients and longitudinal studies examining the predictive power of the procedure.

In conclusion, the recording of TEPs represents a new neurophysiological technique that directly investigates local cortical reactivity and connectivity. TEPs evaluate the cortico-cortical functional connectivity, which is severely impaired in chronic disorders of consciousness [40] and might offer novel contributions to the clinical differentiation between permanent VS and MCS patients. TEPs can be recorded at the patient’s bedside without requiring the collaboration of the patient, and this feature represents a major advantage over structural and functional neuroimaging studies, which require the allocation of considerable technical and financial resources that are not universally available.

## Supporting Information

Figure S1 **The EEG power spectra at the Cz and Pz electrodes.** The FFT spectral analysis does not show statistically significant differences between the minimally conscious state (MCS) and vegetative state (VS) patients (all Fs smaller than 2.19, P>0.79). The red line represents the grand average of the EEG power spectra of the MCS patients, and the black line refers to the VS patients. The dotted lines delineate the frequency bands considered in the analysis.
(TIF)

Material S1 **This file includes FFT spectral analysis performed to verify whether the degree of impairment of the background EEG could differentiate between the two patient groups.**
(DOC)
