# Supplementary figures and images for: Vegetative versus Minimally Conscious States: A Study Using TMS-EEG, Sensory and Event-Related Potentials
Source: PLoS One. 2013 Feb 27;8(2):e57069. doi: 10.1371/journal.pone.0057069 (PMC3584112; doi:10.1371/journal.pone.0057069)

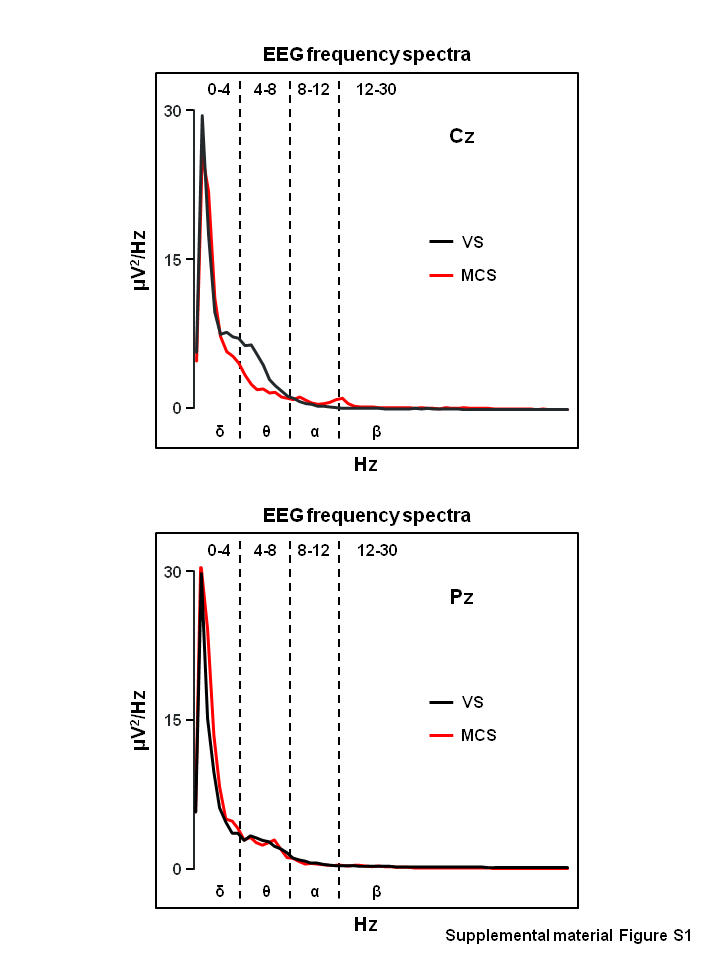

Supplement: Figure S1 — The EEG power spectra at the Cz and Pz electrodes. The FFT spectral analysis does not show statistically significant differences between the minimally conscious state (MCS) and vegetative state (VS) patients (all Fs smaller than 2.19, P>0.79). The red line represents the grand average of the EEG power spectra of the MCS patients, and the black line refers to the VS patients. The dotted lines delineate the frequency bands considered in the analysis. (TIF) [file pone.0057069.s001.tif]
