# Supplementary material for: Vegetative versus Minimally Conscious States: A Study Using TMS-EEG, Sensory and Event-Related Potentials
Source: PLoS One. 2013 Feb 27;8(2):e57069. doi: 10.1371/journal.pone.0057069 (PMC3584112; doi:10.1371/journal.pone.0057069)
Supplement: Material S1 — This file includes FFT spectral analysis performed to verify whether the degree of impairment of the background EEG could differentiate between the two patient groups. (DOC) [file pone.0057069.s002.doc]

**Supplementary material**

**FFT spectral analysis**

We aimed to verify whether the degree of impairment of the background EEG could differentiate between the two patient groups, as suggested by Kotchoubey et al, 2005. Continuous EEG recordings were segmented in epochs of 2 sec. The linked mastoids served as the reference. Epochs containing excessive drift, eye movements, blinks or muscle artefacts were excluded from the analysis (for additional information please see the EEG recordings and analysis in the materials and methods section). The power density was estimated using the Fast Fourier transform (10% Hanning-window; frequency resolution 1 Hz) for all of the frequencies ranging from 0.5 to 45 Hz, divided into five bands as follows: 0.5-4 Hz (delta), 4-8 Hz (theta), 8-12 Hz (alpha), 12-30 Hz (beta) and 30-45 Hz (gamma). The mean band power was then obtained by averaging the power values of all the single-trial epochs for each patient. Statistical analysis (ANOVA) was performed for each frequency band, considering as factors two groups (VS vs. MCS) by nine electrodes (Fz, F3, F4, Cz, C3, C4, Pz, P3 and P4).

The EEG frequency power spectra did no significantly differ between the MCS and VS patients for any of the frequency bands considered in the analysis, all Fs smaller than 2.19, P> 0.79. Data for two electrodes are reported in Figure S1.

Kotchoubey B, Lang S, Mezger G, Schmalohr D, Schneck M, Semmler A, Bostanov V, Birbaumer N. Information processing in severe disorders of consciousness: vegetative state and minimally conscious state. Clin Neurophysiol. 2005 Oct;116(10):2441-53.
